# Relationship between *Blastocystis* infection and clinical outcomes: A scoping review protocol

**DOI:** 10.12688/openreseurope.19281.1

**Published:** 2025-01-20

**Authors:** Varol Tunali, Blin Nagavci, David Carmena, Lucy J. Robertson, Funda Doğruman Al, Eleni Gentekaki, Anastasios D. Tsaousis

**Affiliations:** 1 Department of Microbiology, Izmir University of Economics, Izmir, Turkey; 2 Department of Emergency Medicine, Izmir Metropolitan Municipality Eşrefpaşa Hospital, Izmir, Turkey; 3 Doctoral School of Clinical Medicine, Semmelweis University, Budapest, Budapest, Hungary; 4 Spanish National Centre for Microbiology, Health Institute Carlos III, Parasitology Reference and Research Laboratory, Majadahonda, Madrid, Spain; 5 Centre for Biomedical Research Network in Infectious Diseases (CIBERINFEC), Health Institute Carlos III, Madrid, Spain; 6 Faculty of Veterinary Medicine, Norwegian University of Life Sciences, Ås, Norway; 7 Faculty of Medicine, Department of Medical Microbiology, Division of Medical Parasitology, Gazi University, Ankara, Turkey; 8 Department of Veterinary Medicine, University of Nicosia, Nicosia, Nicosia, Cyprus; 9 School of Natural Sciences, University of Kent, Canterbury, England, UK

**Keywords:** Gut microbiota, Diagnostic methods, Subtype diversity, Intestinal health

## Abstract

*Blastocystis*, a common protist in the human gastrointestinal tract, exhibits substantial genetic diversity and has been linked to varying clinical outcomes. However, its role in human health remains debated, with studies suggesting both commensal and pathogenic interactions. This scoping review aims to systematically map the existing evidence on the association between *Blastocystis* presence and human clinical outcomes. Herein, we present our proposed protocol, where, using systematic search methods, studies will be identified from multiple databases, focusing on diagnostic procedures, clinical outcomes, and treatment options. Findings will provide a comprehensive evidence map, highlighting knowledge gaps and guiding future research. The resulting data is intended to inform clinical and public health perspectives on *Blastocystis* and its potential implications for human health.

## Plain language summary

Blastocystis, a common protist in the human gastrointestinal tract, exhibits substantial genetic diversity and has been linked to varying clinical outcomes. However, its role in human health remains debated, with studies suggesting both commensal and pathogenic interactions. This scoping review aims to systematically map the existing evidence on the association between Blastocystis presence and human clinical outcomes. Herein, we present our proposed protocol, where, using systematic search methods, studies will be identified from multiple databases, focusing on diagnostic procedures, clinical outcomes, and treatment options. Findings will provide a comprehensive evidence map, highlighting knowledge gaps and guiding future research. The resulting data is intended to inform clinical and public health perspectives on Blastocystis and its potential implications for human health

## Introduction

*Blastocystis*, a protist commonly found in the human gastrointestinal tract, is one of the most prevalent eukaryotic microorganisms globally ^1^. It exhibits significant genetic diversity, with at least 44 subtypes (STs) identified to date based on sequence analyses of the small subunit ribosomal RNA ( *SSU* rRNA) gene ^2, 3^. Among these, ST1-ST10, ST12, ST14, ST16, ST23, ST35, and ST41 have been reported from humans, with ST1 to ST4 being the most frequently identified ^4, 5^. The interaction of *Blastocystis* with the gut microbiota and the host immune system has been linked to a range of clinical outcomes, highlighting its potential relevance to human health ^6^.

*Blastocystis* is distributed worldwide, with varying occurrence rates across different regions. It has been reported globally, with variations likely influenced by differences in sanitation and hygiene practices ^7^. *Blastocystis* is found to be present in both healthy individuals and those experiencing gastrointestinal symptoms. Some studies have reported associations between symptoms such as diarrhea, abdominal pain, bloating, and the presence of *Blastocystis* ^8^. However, these associations are inconsistent across studies, and the public health significance of *Blastocystis* remains uncertain, primarily due to conflicting evidence regarding its role in health and disease.

The clinical significance of *Blastocystis* has been a topic of ongoing debate. Historically considered a parasitic organism, some studies have questioned whether certain subtypes or host interactions might contribute to health outcomes in specific contexts ^9^. The genetic diversity of *Blastocystis* adds complexity, with some subtypes being more frequently detected in symptomatic individuals, while others are commonly found in asymptomatic populations. The interplay between *Blastocystis*, the host immune response, and the gut microbiota is believed to play a critical role in determining its impact on human health ^10^.

The existing literature presents diverse perspectives on *Blastocystis*. Some studies suggest that *Blastocystis* presence may be associated with beneficial changes in the gut microbiota, such as increased bacterial diversity and anti-inflammatory responses ^8^. Conversely, some studies report that *Blastocystis* may be associated with reduced beneficial bacteria and an altered gut microbiome composition ^11^. Given the conflicting evidence regarding the pathogenicity of *Blastocystis*, a comprehensive scoping review is warranted to systematically map the existing evidence. This review will synthesize original research studies on human subjects, focusing on the association between *Blastocystis* presence and various clinical outcomes.

### Objectives

The primary objective of this scoping review is to systematically map the existing evidence on *Blastocystis* presence and clinical outcomes in humans. This review will help clarify the current state of knowledge, identify research gaps, and provide a foundation for future studies that can further explore this relationship. The following research questions will be assessed:

1. What evidence exists on the relationship between *Blastocystis* occurrence and human clinical outcomes?
2. What evidence exists regarding the diagnostic methods used for *Blastocystis* detection, and their performance and accuracy?
3. What evidence is available on the treatment and management options for *Blastocystis*?

## Methods

This scoping review will be conducted and reported in accordance with the Preferred Reporting Items for Systematic reviews and Meta-Analyses (PRISMA) extension for scoping reviews (PRISMA-ScR) ^12^.

### Literature searches

Systematic searches for relevant published studies will be conducted in PubMed, EMBASE, Web of Science, and the Cochrane Library. An experienced methodologist will develop and validate search strategies to ensure maximum sensitivity. Searches will be performed without time restrictions, and the date of the search, search strategy, and number of search results will be documented for each database.

### Selection of studies

The selection process will involve two stages: screening and eligibility assessment. Titles and abstracts will be screened by two independent reviewers to identify potentially relevant studies, using Rayyan. Full-text articles of studies deemed relevant will be retrieved and assessed for eligibility based on predefined criteria, by two reviewers. Disagreements will be resolved through discussion or by consulting a third reviewer. Screening results and reasons for exclusion will be documented in PRISMA flowcharts.

### Eligibility criteria

To be included in this scoping review, studies must involve human participants with confirmed (or, for the diagnostic question, suspected) *Blastocystis* infection. To be a confirmed case, the infection must be diagnosed using reliable methods such as direct microscopy, culture, conventional and qPCR, or next-generation sequencing (NGS). Studies that primarily focus on other parasitic infections, where *Blastocystis* is not the main focus, will be excluded.

Studies that include both symptomatic and asymptomatic individuals with *Blastocystis* infection will be eligible, as this should assist in providing a comprehensive understanding of the organism’s potential pathogenicity and its association with various clinical outcomes ( Table 1).

**Table 1.** Inclusion and exclusion criteria for each research question.

	Research Question	Population	Exposure	Comparator	Outcomes	Study Design	Exclusion criteria
1	What evidence exists on the relationship between Blastocystis infection and clinical outcomes, and pathogenicity in humans?	Humans of all ages	*Blastocystis* infection	No *Blastocystis* infection	All reported clinical outcomes (e.g., gastrointestinal/extra- intestinal symptoms, pathogenicity etc.)	Cross-sectional, cohort studies, case-control studies, prevalence studies, systematic reviews.	**Study Type**: Editorials, letters, commentaries, conference abstracts, case series, case reports, clinical guidelines, narrative reviews (though reference lists will be screened), and non-peer- reviewed works (e.g., theses, preprints). **Subject**: Animal studies or research conducted solely in non-human settings (e.g., laboratory/ animal models). **Confirmation**: Studies where Blastocystis infection is neither confirmed nor suspected. Results: Studies reporting combined treatment outcomes without *Blastocystis*-specific results **Focus**: Studies where *Blastocystis* is not the main focus, including those primarily addressing other parasitic infections or treatments not targeting *Blastocystis* specifically. **Language**: Except English, Greek, Spanish, German, French, Norwegian, Danish, Russian, and Turkish.
	Research Question	Population	Index Test	Reference Test	Outcomes	Study Design	
2	What evidence exists regarding the diagnostic methods used for *Blastocystis* diagnosis, and their performance?	Humans of all ages suspected of Blastocystis infection	Diagnostic methods (e.g., microscopy, culture, molecular diagnostics)	Any reference standard or another diagnostic method	All reported test accuracy outcomes.	Diagnostic accuracy studies, clinical trials, systematic reviews.	
	Research Question	Population	Intervention	Comparator	Outcomes	Study Design	
3	What evidence is available on the treatment options for *Blastocystis* infection, and how effective are these interventions in managing the infection and associated symptoms?	Humans of all ages with confirmed *Blastocystis* infection	Any treatment for *Blastocystis*	No treatment, placebo, or alternative strategies	All reported outcomes (e.g. eradication of *Blastocystis*, symptom resolution etc.)	Clinical trials, cross- sectional, cohort studies, case-control studies, systematic reviews.	

Studies published in all languages spoken by the panel members will be considered (English, Greek, Spanish, German, French, Norwegian, Danish, Russian, and Turkish), in order to maximize data capture.

Eligible study types include randomized and non-randomized trials, cohort studies, case-control, systematic reviews, and cross-sectional studies (with or without a control group). Editorials, letters, commentaries, conference abstracts, case series, case reports, clinical guidelines, narrative reviews, and non-peer-reviewed work (e.g. theses), will be excluded. Narrative review articles will not be included, but their reference lists will be explored for further data sources ( Table 1).

No date limit will be used for the inclusion criteria, in order to provide a full evidence map on this topic.

### Data extraction

Data from included studies will be charted using a standardized data extraction form. The form will be piloted to ensure consistency and reliability. Data extraction will be performed independently by two reviewers, with cross-checking to resolve discrepancies. Additional data or clarifications will be sought from study authors when required.

### Data items

The data items to be extracted will include:

- Study Characteristics: Author, year of publication, study type, design, setting, and geographical location.
- Population Details: Number of participants, age, gender
- Details of *Blastocystis* Infection: Detection methods used, subtype identification, presence or absence of symptoms.
- Clinical Outcomes: All reported symptoms (including gastrointestinal, dermatologic etc. symptoms), associations with *Blastocystis* subtypes (if reported), any given treatment and treatment outcome, and any other clinically relevant outcome
- Subgroups: studies will be presented in subgroups based on study design and patient population.

## Results

The results section will describe the evidence of the relationship between *Blastocystis* infection and clinical outcomes, variations in outcomes based on diagnostic methods, and the pathogenicity of specific *Blastocystis* subtypes. Identified and included studies will be presented in both tabular and graphical forms, offering a complete evidence map on this topic. Research gaps will also be identified, and recommendations for future studies will be provided. 

### Critical appraisal of individual sources of evidence

As critical appraisal is not a mandatory step in scoping reviews, it is not planned to be conducted.

## Discussion and conclusions

The findings of this scoping review will provide a comprehensive synthesis of the current evidence on *Blastocystis's* potential associations with clinical outcomes. Despite its high prevalence and global distribution, *Blastocystis* remains poorly understood, with conflicting perspectives regarding its clinical relevance ^13^.

In conclusion, this scoping review aims to serve as a foundational resource for researchers and clinicians, guiding the design of targeted studies and providing a robust evidence base for *Blastocystis* research.

## Ethics and consent

Ethics and consent were not required.

## Data Availability

No data are associated with this article
