# Supplementary material for: Relationship between Blastocystis infection and clinical outcomes: A scoping review protocol
Source: Open Res Eur. 2025 Jan 20;5:15. [Version 1] doi: 10.12688/openreseurope.19281.1 (PMC11862357; doi:10.12688/openreseurope.19281.1)
Supplement: Peer review history

# Reviewer report: Reviewer response for version 1

**DOI:** 10.21956/openreseurope.20870.r50449

**Published:** 2025-03-03

**Authors:** Renzo S Salazar-Sánchez

**Affiliations:** 1 Heidelberg University Hospital Institute of Medical Biometrics (Ringgold ID: 222689), Heidelberg, Baden-Württemberg, Germany

This study protocol, entitled "Relationship between *Blastocystis* Infection and Clinical Outcomes: A Scoping Review Protocol," is a well-structured and meticulously designed proposal. It outlines a standardized approach to conducting a systematic review across multiple databases, with a particular focus on *Blastocystis,* a globally distributed protozoan whose role in human health remains uncertain. The primary objective of this review is to establish a comprehensive understanding of the existing evidence concerning its clinical implications in humans.

 This proposal aims to address three critical research questions supported by scientific evidence related to:

     1. The association between *Blastocystis* occurrence and human clinical outcomes.

     2. The diagnostic methodologies employed for *Blastocystis* detection, including their performance and accuracy.

     3. The availability and efficacy of treatment and management strategies for *Blastocystis *infections.

 Given the controversial nature and complex biological characteristics of *Blastocystis*, it is imperative to synthesize and critically appraise the available literature. This will facilitate a comprehensive and evidence-based understanding of its clinical significance.

 Methodological Considerations

 The proposed protocol delineates a logical and well-defined workflow, incorporating key methodological criteria to ensure the quality and reliability of the findings. However, I would like to highlight two potential limitations that were not explicitly addressed in the methodology and could introduce bias into the conclusions:

     1. Literature Search Strategy: The authors indicate that systematic searches will be conducted using PubMed, EMBASE, Web of Science, and the Cochrane Library. To enhance the breadth and comprehensiveness of the review, I suggest including Scopus and SciELO to provide broader coverage, particularly for Latin American and regional studies.

     2. Eligibility Criteria: The inclusion criteria define eligible studies as those involving human participants with confirmed *Blastocystis *infection, irrespective of symptomatology (symptomatic or asymptomatic). However, the protocol does not clearly outline how studies involving multiple *Blastocystis* subtypes, cases of mixed infections, or coinfections with other commensal protozoa will be managed. This is particularly relevant given the prevalence of coinfections reported in Latin American studies. Such polyparasitic infections can contribute to symptomatology, making it essential to address this aspect explicitly to avoid misinterpretations in the findings.

 Table 1 provides a clear and coherent summary of the study design and key points.

 Conclusion

 In conclusion, this study protocol represents a valuable and well-founded initiative to address a complex and debated issue regarding *Blastocystis*. Given its global distribution and potential clinical relevance, a rigorous and systematic literature assessment is essential.

 Therefore, I endorse this study protocol and recommend its approval, with the suggested refinements to further strengthen its methodological robustness.

Is the study design appropriate for the research question?

Yes

Is the rationale for, and objectives of, the study clearly described?

Yes

Are sufficient details of the methods provided to allow replication by others?

Yes

Are the datasets clearly presented in a useable and accessible format?

Not applicable

Reviewer Expertise:

Infectious diseases, Molecular biology, Microbiome

I confirm that I have read this submission and believe that I have an appropriate level of expertise to confirm that it is of an acceptable scientific standard.

# Reviewer report: Reviewer response for version 1

**DOI:** 10.21956/openreseurope.20870.r50442

**Published:** 2025-02-26

**Authors:** Supaluk Popruk

**Affiliations:** 1 Mahidol University, Bangkok, Thailand

This study offers valuable information for conducting a systematic review and meta-analysis. Additionally, the methodology outlined can guide future studies in replicating or expanding upon the results. Furthermore, the study can serve as a foundation for evidence-based data, which can inform future research directions and can be the guidelines for other parasites or pathogens. The article exhibits a notable weakness in that it lacks a clear delineation of participant types, such as children, villagers, individuals with close contact to animals, and caregivers. The inclusion of these distinct groups may have led to different outcomes.

 However, the strengths of this article are guidelines of the related research in systematic review and meta-analysis.

Is the study design appropriate for the research question?

Yes

Is the rationale for, and objectives of, the study clearly described?

Yes

Are sufficient details of the methods provided to allow replication by others?

Yes

Are the datasets clearly presented in a useable and accessible format?

Yes

Reviewer Expertise:

I work on parasitology.

I confirm that I have read this submission and believe that I have an appropriate level of expertise to confirm that it is of an acceptable scientific standard.

# Reviewer report: Reviewer response for version 1

**DOI:** 10.21956/openreseurope.20870.r50447

**Published:** 2025-02-25

**Authors:** Arutchelvan Rajamanikam

**Affiliations:** 1 University Malaya, Kuala Lumpur, Malaysia

The article is timely to systematically review the literature on the complex and controversial subject of  clinical outcome due to *Blastocystis*. This scoping review is concise and all encompassing. However, there could be more evidence on pathogenic characteristics in *Blastocystis*, based on previous studied could have been included. 

 Otherwise, the write-up is clear and serves its purpose.

Is the study design appropriate for the research question?

Yes

Is the rationale for, and objectives of, the study clearly described?

Yes

Are sufficient details of the methods provided to allow replication by others?

Yes

Are the datasets clearly presented in a useable and accessible format?

Not applicable

Reviewer Expertise:

Diagnostic Parasitology, Host-parasite interaction

I confirm that I have read this submission and believe that I have an appropriate level of expertise to confirm that it is of an acceptable scientific standard.

# Reviewer report: Reviewer response for version 1

**DOI:** 10.21956/openreseurope.20870.r50443

**Published:** 2025-02-14

**Authors:** Małgorzata Lepczyńska

**Affiliations:** 1 University of Warmia and Mazury, Żołnierska, Poland

The manuscript is written clearly and is easy to understand. It presents a scoping review protocol, focusing on literature searching strategy for clarifying the current state of knowledge, identifying research gaps, and guiding future research in understanding the relationship between *Blastocystis* presence and clinical outcomes in humans.

 The introduction of the manuscript highlights the complexity and analytical challenges of determining *Blastocystis* pathogenicity as well as the sensitivity of detection methods in order to address the conflicting evidence regarding symptomatic and asymptomatic human intestine colonization. The authors underline the high genetic diversity of *Blastocystis*—up to 44 subtypes (STs). Each subtype may have a different, direct or indirect, influence on human health (e.g., through various interactions with the gut microbiota). The interplay between *Blastocystis* subtypes, the host immune response, and the gut microbiota is believed to play a critical role in determining its impact on human health.

 According to the available literature, it is impossible to state clearly whether *Blastocystis* is a pathogen or a commensal/beneficial organism to the human gut. In the paper, the authors provided a protocol for conducting a scoping review to assess which data should be taken into consideration in case of performing a review of all accessible studies on human subjects, focusing on the association between *Blastocystis* presence and various clinical outcomes.

 The scoping review protocol clearly describes and explains the purpose of the selection process of published research regarding the evidence on the relationship between *Blastocystis* occurrence and human disease, the performance and accuracy of diagnostic methods used for *Blastocystis* detection, and the treatment and management options for *Blastocystis*. The manuscript presents the assumptions of the proposed protocol in an accessible and concise way, emphasizing the importance of the systematic search methods.

 The protocol adequately explained, point by point, defined criteria of inclusion and exclusion of published articles, essential for the given objectives. I consider this protocol extremely important. There are a lot of scientific articles in the recent literature; however, many of them do not provide reliable results—basing on a small study population (or case reports), reporting the patients symptoms without any confirmation of a physician (as *Blastocystis* infection has been associated with non-specific gastrointestinal symptoms such as abdominal pain, diarrhea, nausea, vomiting, bloating, and anorexia, we actually are not able to say for sure if it is the only cause of reported symptoms), or studies where *Blastocystis* is not the main focus, targeting other parasitic infections and accidentally detecting *Blastocystis*, etc. Additionally, the papers containing the methodology of *Blastocystis* detection, which is based on microscopy, culture, or ELISA tests only, in times of developed molecular techniques giving the subtype distribution results, are not enough.

 The authors rightly highlight the crucial importance of findings that will provide a comprehensive evidence map, providing future study in understanding the nature of *Blastocystis* (helping to unravel what factors determine asymptomatic colonization or symptomatic disease), thereby clearly demonstrating the appropriateness of creating a schedule of literature searching.

 The authors pointed to the databases that will be searched and clearly defined the studies selection process (screening and eligibility assessment by two or three reviewers), as well as the eligibility criteria (in the table). At the end, the authors also clarify how the proposed protocol will identify and fill possible gaps in existing knowledge, and according to that, recommendations for future studies will be provided. As a result of the scoping review according to the created protocol, identified and included studies will be presented in two different (tabular and graphical) forms, offering a complete evidence map on the mentioned topic.

Is the study design appropriate for the research question?

Yes

Is the rationale for, and objectives of, the study clearly described?

Yes

Are sufficient details of the methods provided to allow replication by others?

Yes

Are the datasets clearly presented in a useable and accessible format?

Not applicable

Reviewer Expertise:

medical parasitology (mainly Blastocystis), microbiology, medical biology,medical education

I confirm that I have read this submission and believe that I have an appropriate level of expertise to confirm that it is of an acceptable scientific standard.
